# Quality of Sleep in the Cypriot Population and Its Association With Multimorbidity: A Cross-Sectional Study

**DOI:** 10.3389/fpubh.2021.693332

**Published:** 2021-10-29

**Authors:** Maria Kyprianidou, Demosthenes Panagiotakos, Maria Kambanaros, Konstantinos C. Makris, Costas A. Christophi

**Affiliations:** ^1^Cyprus International Institute for Environmental and Public Health, Cyprus University of Technology, Limassol, Cyprus; ^2^Department of Nutrition and Dietetics, School of Health Sciences and Education, Harokopio University, Athens, Greece; ^3^Department of Allied Health and Human Performance, University of South Australia, Adelaide, SA, Australia

**Keywords:** sleep, quality of sleep, multimorbidity, chronic disease, Cyprus

## Abstract

Poor sleep is a relatively common condition with possibly serious adverse health consequences. Lack of sleep affects the endocrine, immune, and nervous systems. In Cyprus, there is no information about the quality of sleep in the population. The goal of this study was to assess the quality of sleep in the Cypriot population and evaluate its association with multimorbidity. A representative sample of the adult population of Cyprus was selected in 2018–2019 among the five government-controlled municipalities of the Republic of Cyprus using stratified sampling. Data on sleep quality as well as on the presence of chronic, clinical, and mental health conditions were collected using a validated questionnaire. Diseases were classified according to the International Classification of Diseases, 10th Revision (ICD-10). A total of 1,140 Cypriot men and women over 18-years of age (range: 18–94) participated in the study. The median Pittsburgh sleep quality index score of the participants was 5 (first quartile = 3, third quartile = 7) with the maximum score being 17, which suggests that the Cypriot population has a relatively good quality of sleep overall, although, almost one-third of the study population had a poor quality of sleep. Women, residents of Paphos, and married people had a poorer quality of sleep (*p* < 0.05). Having a poor quality of sleep was associated with higher odds of multimorbidity (OR = 2.21, 95% CI: 1.55, 3.16), even after adjusting for demographics, socioeconomic, and lifestyle factors. Adopting good sleep habits could be beneficial and would potentially help reduce the risk of multimorbidity. Public health guidelines regarding the importance of sleep and its association with multimorbidity should be considered.

## Introduction

Poor sleep is a major public health concern and a common medical condition with serious adverse consequences (1). Sufficient sleep enhances memory (2) and is associated with self-rated happiness (3), and with good academic performance (2, 4). On the other hand, poor sleep affects the endocrine, immune, and nervous systems (5). It also increases cardio-metabolic risk as it is associated with obesity, diabetes, impaired glucose tolerance, and hypertension (6, 7).

Several studies have reported a high prevalence of sleep disturbances among older adults (8–12). Specifically, among French-speaking adults in the province of Quebec (Canada), 25.3% were dissatisfied with their sleep, 29.9% had symptoms of insomnia, and about 10% reported suffering from an insomnia syndrome (9). In contrast, based on cross-sectional Cooperative Health Research in the Region of Augsburg Age Study, 8.2% were classified as having insomnia in Germany (12). Aging is related to changes in sleep; specifically, total sleep time and sleep efficiency decrease with aging. In addition, the time one is awake after sleep onset, the number of arousals from sleep, and sleep latency increases (13). However, insufficient sleep is not only common among older people but in younger age groups too (14). It is reported that younger people have difficulties with their sleep (15) due to several factors including prolonged working hours and irregular sleep schedules (6), and also the use of electronics in daily life. Socioeconomic factors (16, 17), physical activity (18), dietary habits (19, 20), and other physiological and psychosocial factors (16) can affect sleep quality and are associated with common sleep disorders. Sleep disturbances have serious adverse consequences (1) that are associated with several chronic diseases, such as type 2 diabetes (21), cardiovascular disease (22, 23), and depression (24). Moreover, quality of sleep is affected by sleep disorders, such as obstructive sleep apnea (6).

In recent years, multimorbidity, which is defined as the coexistence of two or more chronic conditions (25), has increased significantly (26). The overall prevalence of multimorbidity in adults has been reported as 18–30% (27, 28), and in Cyprus, the age and gender-standardized prevalence of multimorbidity is estimated to be 29% (29), which is similar to the prevalence in other countries, such as Scotland (23.2%) (30), Australia (25.5%) (31), Serbia (26.9%) (32), and Brazil (23.6%) (33). Several studies (34–36) have shown an association between sleep disorders and multimorbidity. Short sleep duration was associated with an increase in the number of chronic conditions in an individual (34). Moreover, an epidemiological cross-sectional study reported associations between insomnia and multimorbidity in elderly people (35) and argued that people diagnosed with insomnia had poorer physical health. It is also reported that patients with obstructive sleep apnea present with multimorbidity (36). To the best of our knowledge, there is no data on the quality of sleep of the Cypriot population; however, a recent cross-sectional study (27) reported that the proportion of people with intermediate to high risk for obstructive sleep apnea was 50% in men and 18% in women in the general population. Given the lack of information on the quality of sleep in Cyprus and the importance of sleep in a specific population as a crucial public health issue as well as the clinical significance of multimorbidity, there were two objectives of the study: (1) to assess the quality of sleep in the Cypriot population and (2) to examine the relationship between multimorbidity and quality of sleep in the adult general population.

## Methods

### Study Design

This was a cross-sectional study.

### Setting

Data collection occurred in public areas (e.g., restaurants, malls, cafes, and village squares) and houses throughout Cyprus, between May 2018 and June 2019. The study included men and women aged over 18 years (*n* = 1,140), living in the five government-controlled municipalities of the Republic of Cyprus (Nicosia, Limassol, Larnaca, Paphos, and Ammochostos). A stratified sampling procedure was implemented to ensure that the sample matched the Cypriot population in three key demographic characteristics, namely age, sex, and geographical region. To confirm that the sample matched the Cypriot population in the key characteristics considered in the study, we performed chi-square goodness of fit tests comparing the distribution of the sample size for each key characteristic and the corresponding distribution in the overall Cypriot population. The selected study sample was similar to the general population with respect to distributions of the region, age, and gender (all *p* > 0.05).

### Ethics Approval

The study was conducted according to the Declaration of Helsinki guidelines, and all procedures involving research study participants were approved by the Cyprus National Bioethics Committee (CNBC) (EEBK EΠ 2018.01.123). The application submitted to CNBC outlined the study objectives and outcomes, the data collection process and the data management, the use of the data and the expected benefits, and included the questionnaire of the study. All the details of the study were also provided on the first page of the questionnaire. Trained researchers approached potential participants, explained the purpose of the study, and informed them that participation was anonymous and that they could stop participating at any time.

### Characteristics of Participants

Data included sociodemographic characteristics (e.g., age in years, gender, marital status, educational status, and monthly income), lifestyle habits (e.g., current smoking and physical activity), body mass index (BMI), data on quality of life, and a detailed medical history. Medical history was collected through a face-to-face interview using a standardized questionnaire.

Marital status was reported as single, married/engaged, or separated/-divorced/widowed. Education level was classified into three categories commonly used in Cyprus and similar to a study on the Greek population (25), namely: (i) primary education (participants who completed only primary school, <7 years of schooling); (ii) secondary education (participants who completed middle or high school, 7–12 years of schooling); and (iii) higher education (participants who have a university degree, >12 years of schooling). Financial status was described using the annual income and was classified as (i) low (≤€6,500 per year); (ii) middle (€6,500–19,500 per year); and (iii) high (>€19,500 per year). Current smokers were those who smoked at least one cigarette a day and not current smokers included never and former smokers. BMI was calculated as weight (in kilograms) divided by standing height (in meters) squared. Obesity was then defined as BMI > 29.9 kg/m^2^, overweight as BMI 25–29.9 kg/m^2^, normal as BMI 18.5–24.9 kg/m^2^, and underweight as BMI <18.5 kg/m^2^, according to the WHO classification. The quality of life section included the EQ-5D-3L questionnaire (Greek version) (25).

### Medical Assessment

The medical history section of the questionnaire included 47 chronic diseases of all the human systems (e.g., circulatory, digestive/excretory, endocrine, immune, nervous, renal/urinary, reproductive, respiratory, and skeletal/muscular systems, and neoplasms) which were coded according to the International Classification of Diseases (ICD-10). This section of the questionnaire was completed by the researchers *via* a face-to-face interview, using the question “Have you ever been diagnosed by a physician with any of the following chronic diseases? Choose all that apply.” Multimorbidity was defined “as any combination of two or more chronic conditions in an individual” (37).

### Quality of Sleep Assessment

Quality of sleep was assessed using the Greek-version of the Pittsburgh sleep quality index (PSQI) questionnaire (38), kindly provided by the University of Pittsburgh. The PSQI consists of 19 self-rated questions [an additional five questions that are rated by the bed-partner or roommate, which are used for clinical information only and are not part of the calculation of the PSQI score, were not used in this study]. The 19 self-rated questions assess a wide variety of factors relating to sleep quality, including estimates of sleep duration and latency, and the frequency and severity of specific sleep-related problems. These 19 items are grouped into seven components (subjective sleep quality, sleep latency, sleep duration, habitual sleep efficiency, sleep disturbances, use of sleeping medications, and daytime dysfunction), each scored on a 0–3 scale [minimum score = 0 (better); maximum score = 3 (worse)]. The seven-component scores are then summed to yield a global PSQI score, which has a maximum score of 21, with higher scores indicating worse sleep quality.

### Statistical Analysis

Continuous variables (such as age, weight, height, BMI, and quality of life score) are presented as mean ± standard deviation (SD) and categorical variables (such as age group, gender, geographical area, residency, marital status, educational status, salary category, physical activity level, smoking, obesity group, and quality of sleep levels) as absolute and relative (%) frequencies. The tertiles of the quality of sleep were defined as follows: good quality of sleep (score ≤ 4), moderate quality of sleep (score 5–6), and poor quality of sleep (score ≥ 6) to split the distribution of the quality of sleep score into three parts, each containing a third of the study population. We also grouped the quality of sleep score based on the original paper of the PSQI development (38), which indicates that a global PSQI score greater than 5 was a sensitive and specific measure for the poor quality of sleep as related to clinical and laboratory measures.

The distribution of continuous variables was assessed for normality first and associations between normally distributed variables and the quality of sleep score tertiles were evaluated using the one-way analysis of variance. For non-normally distributed variables, the Kruskal–Wallis test was used instead. The chi-squared test was applied to evaluate potential associations between categorical variables and quality of sleep tertiles.

Logistic regression models were used to examine the association of quality of sleep (in tertiles and separately as scores >5 vs. ≤ 5) with the probability of having multimorbidity (yes/no) after adjusting for possible confounders, including age, gender, smoking, and physical activity status. Model 1 was the unadjusted model, Model 2 was the model adjusted for demographic (age, gender) and socioeconomic characteristics (educational status, marital status, salary group, geographical area, and residency), and Model 3 was further adjusted for lifestyle characteristics (smoking status and physical activity level). Interaction terms between the quality of sleep tertiles and age as well as gender were tested using the Wald test and in the case of significant interactions, a stratified analysis was conducted. The fit of the model was assessed using the Hosmer–Lemeshow goodness of fit test.

Statistical analysis was conducted using STATA 14.0 statistical software (Stata Corp, College Station, TX, USA). All statistical hypotheses were two-sided, and tests with a *p* < 0.05 were deemed statistically significant.

## Results

### Characteristics of the Participants

A total of 1,140 people participated in the study among whom 642 (56.4%) were women. The mean age of the participants was 41 ± 17 years (40 ± 16 years among women and 42 ± 18 years among men). The majority of the participants were residents of urban regions (76.3%), 54% were married, 64% had completed higher education, about half of them had a yearly average salary of €6,500–19,500, and 40% were private employees. About 35% of the individuals were current smokers, and among them, 58% were men, whereas about 50% of the study participants were physically active. In addition, the overall mean BMI was 25.0 ± 5.0 kg/m^2^, with 24.9 ± 4.8 kg/m^2^ among women and 26.4 ± 4.0 kg/m^2^ among men. We observed a statistically significant difference in the physical activity level between the different age groups. Specifically, among younger people (18–44 years old) the majority exercised regularly, whereas among those who were 65 years old and older only 30% exercised regularly. Moreover, we found a higher mean quality of life score in people 18–64 years old as opposed to elderly people (*p* < 0.01) (Supplementary Table 1).

### Multimorbidity

The overall age and gender standardized prevalence of multimorbidity, as previously reported (29) was 28.6% (95% CI: 26.0, 31.2). The mean number of conditions was 1.07. The most prevalent chronic diseases were the diseases of the circulatory system, followed by the endocrine system and the digestive–excretory system, whereas the most common combinations among people with multimorbidity were diseases of the circulatory and endocrine systems (25%), followed by circulatory and digestive–excretory systems (20%), and circulatory and nervous systems (19%) (29).

### Quality of Sleep

The median PSQI score of the participants was five (first quartile = 3, third quartile = 7) with the maximum score being 17. We found a statistically significant difference between men and women in terms of the quality of sleep tertiles (Table 1). A higher proportion of men was in the good sleep tertile compared with the corresponding percentage among women (50.5 vs. 45.0%, respectively) and a lower proportion of men was in the poor sleep tertile compared with the corresponding percentage among women (24.5 vs. 31.8%, respectively) (*p* = 0.03). Similarly, a higher proportion of the residents of the Pafos area compared with other municipalities (*p* < 0.01) and of divorced/widowed people compared with married or single participants (*p* = 0.03) were in the poor quality of sleep tertile (Table 1). Based on the categorization of quality of sleep score as good (score ≤ 5) and poor (score > 5) sleepers, statistically significant associations were revealed for gender and geographical areas with being good or poor sleeper (Supplementary Table 2).

**Table 1:** Demographics, socio-economic and lifestyle characteristics overall and by quality of sleep tertiles.

	**Quality of sleep**				
**Characteristics**	**Overall^a^**	**Good^d^**	**Moderate^d^**	**Poor^d^**	***P*** **-value**
	**(***N*** = 1,140)**	**(***N*** = 541)**	**(***N*** = 273)**	**(***N*** = 326)**	
**Quality of sleep score** ^a^	5 (3, 7)	3 (2, 4)	5 (5, 6)	9 (7, 10)	**<0.01** ^b^
**Number of morbidities**	1 (0, 2)	0 (0, 1)	1 (0, 1)	1 (1, 2)	**<0.01** ^b^
**Age group**					
18–24	5 (3, 7)	76 (45.5)	49 (29.3)	42 (25.2)	0.17^c^
25–44	5 (3, 7)	247 (47.1)	135 (25.8)	142 (27.1)	
45–64	5 (3, 7)	155 (49.4)	62 (19.7)	97 (30.9)	
65+	5 (3, 8)	63 (46.7)	27 (20.0)	45 (33.3)	
**Sex**					
Male	4 (3, 6)	251 (50.5)	124 (25.0)	122 (24.5)	**0.03** ^c^
Female	5 (3, 7)	289 (45.0)	149 (23.2)	204 (31.8)	
**Geographical area**					
Nicosia	5 (3, 7)	239 (48.5)	124 (25.1)	130 (26.4)	**<0.01** ^c^
Limassol	5 (3, 7)	151 (48.5)	72 (23.2)	88 (28.3)	
Larnaka	5 (3, 7)	83 (48.5)	43 (25.2)	45 (26.3)	
Paphos	6 (4, 8)	42 (37.2)	19 (16.8)	52 (46.0)	
Ammochostos	5 (2, 6)	24 (48.0)	15 (30.0)	11 (22.0)	
**Residency**					
Urban	5 (3, 7)	408 (47.2)	202 (23.4)	254 (29.4)	0.54^c^
Rural	5 (3, 7)	128 (47.6)	80 (26.0)	71 (26.4)	
**Marital status**					
Married	5 (3, 7)	307 (49.8)	130 (21.1)	179 (29.1)	**0.03** ^c^
Single	5 (3, 7)	185 (43.9)	123 (29.2)	113 (26.9)	
Divorced/Widowed	5 (3, 8)	47 (49.0)	18 (18.7)	31 (32.3)	
**Educational status**					
Primary	5 (3, 8)	30 (45.5)	13 (19.7)	23 (34.8)	0.79^c^
Secondary	5 (3, 7)	163 (48.2)	82 (24.3)	93 (27.5)	
Higher	4 (1, 6)	343 (47.0)	177 (24.3)	209 (28.7)	
**Salary group**					
Low	5 (3, 7)	104 (43.2)	64 (26.5)	73 (30.3)	0.19^c^
Middle	5 (3, 7)	259 (46.1)	140 (24.9)	163 (29.0)	
High	4 (3, 7)	173 (52.7)	68 (20.7)	87 (26.6)	
**Physically active**					
Yes	4 (3, 7)	275 (50.8)	125 (23.1)	141 (26.1)	0.08^c^
No	5 (3, 7)	262 (44.3)	147 (24.9)	182 (30.8)	
**Current smoker**					
Yes	5 (3, 7)	178 (44.3)	97 (24.1)	127 (31.6)	0.18^c^
No	5 (3, 7)	360 (49.3)	175 (23.9)	196 (26.8)	
**BMI group**					
Underweight	5 (3, 7)	18 (42.9)	13 (31.0)	11 (26.1)	0.15^c^
Normal	5 (3, 7)	277 (49.0)	142 (25.2)	146 (25.8)	
Overweight	5 (3, 8)	164 (45.3)	75 (20.7)	123 (34.0)	
Obese	5 (3, 7)	75 (49.3)	38 (25.0)	39 (25.7)	

*Bold values represent statistically significant associations p < 0.05; BMI (body mass index)*.

a *Median PSQI score (first quartile, third quartile)*.

b *Kruskal-Wallis equality-of-populations rank test*.

c *Pearson's chi-squared test*.

d *N (%)*.

Even though, the median score of the quality of sleep was higher in the group who completed primary or secondary education compared to those who completed a secondary or a higher education, among individuals who had a low or middle average yearly salary and in physically inactive people compared with individuals who had a middle or high average yearly salary and physically active participants, respectively, these differences were not statistically significant (*p* > 0.05). Furthermore, other characteristics, such as age groups, urban vs. rural, smoking, and BMI groups were similar among the three tertiles of quality of sleep (Table 1).

Table 2 presents the PSQI score components by the different characteristics of the participants. Although there were no large differences in any of the PSQI score components, it is noted that, in the majority of the components, statistically significant differences among the age groups were observed. Specifically, the worst sleep duration was found in participants aged 45–64 years (median score = 1, first quartile = 0, third quartile = 2), whereas most sleep disturbances were found in the older group (median score = 1, first quartile = 1, third quartile = 2) (*p* < 0.01). Furthermore, individuals who were single, and those who had a normal BMI, had a longer sleep duration compared with married/divorced/widowed participants (*p* < 0.01) and with overweight/obese/underweight people (*p* < 0.01), respectively. On the other hand, people who had a high annual income had a shorter sleep duration compared with those who had a low or a middle annual income (*p* < 0.01). We did not find any statistically significant differences in any of the PSQI score components between residents of urban and rural regions and between smokers and non-smokers.

**Table 2:** Demographics, socio-economic and lifestyle characteristics by the PSQI components.

	**PSQI components**													
**Characteristics**	**Sleep**	***P*** **-value**	**Sleep**	***P*** **-value**	**Sleep**	***P*** **-value**	**Daytime**	***P*** **-value**	**Sleep**	***P*** **-value**	**Sleep**	***P*** **-value**	**Sleep**	***P*** **-value**
	**duration**		**disturbances**		**latency**		**dysfunction**		**efficiency**		**medication**		**quality**	
**Age group**														
18–24	0 (0, 1)	**<0.01** ^b^	1 (1, 1)	**<0.01** ^b^	1 (0, 2)	0.06^b^	1 (0, 1)	**<0.01** ^b^	0 (0, 1)	0.02^b^	0 (0, 0)	0.25^b^	2 (2, 2)	**0.01** ^b^
25–44	0 (0, 1)		1 (1, 1)		1 (0, 2)		1 (0, 1)		0 (0, 1)		0 (0, 0)		2 (1, 2)	
45–64	1 (0, 2)		1 (1, 1)		1 (0, 2)		1 (0, 1)		0 (0, 1)		0 (0, 0)		2 (1, 2)	
65+	0 (0, 1)		1 (1, 2)		1 (0, 2)		0 (0, 1)		0 (0, 1)		0 (0, 0)		2 (1, 2)	
**Sex**														
Male	0 (0, 1)	0.90^a^	1 (1, 1)	0.33^a^	1 (0, 1)	**0.05** ^a^	1 (0, 1)	**<0.01** ^a^	0 (0, 1)	0.17^a^	0 (0, 0)	1.00^a^	2 (1, 2)	0.68^a^
Female	0 (0, 1)		1 (1, 1)		1 (0, 2)		1 (0, 1)		0 (0, 1)		0 (0, 0)		2 (1, 2)	
**Geographical area**														
Nicosia	0 (0, 1)	0.77^b^	1 (1, 1)	0.80^b^	1 (0, 2)	**0.01** ^b^	1 (0, 1)	0.22^b^	0 (0, 1)	0.08^b^	0 (0, 0)	0.63^b^	2 (1, 2)	**<0.01** ^b^
Limassol	0 (0, 1)		1 (1, 1)		1 (0, 2)		1 (0, 1)		0 (0, 1)		0 (0, 0)		2 (1, 2)	
Larnaka	0 (0, 1)		1 (1, 1)		1 (0, 2)		1 (0, 1)		0 (0, 1)		0 (0, 0)		2 (1, 2)	
Paphos	0 (0, 2)		1 (1, 1)		1 (0, 2)		1 (0, 1)		0 (0, 1)		0 (0, 0)		2 (2, 3)	
Ammochostos	1 (0, 1)		1 (1, 1)		1 (0, 2)		0.5 (0, 1)		0 (0, 1)		0 (0, 0)		2 (1, 2)	
**Residency**														
Urban	0 (0, 1)	0.96^a^	1 (1, 1)	0.67^a^	1 (0, 2)	1.00^a^	1 (0, 1)	0.69^a^	0 (0, 1)	1.00^a^	0 (0, 0)	1.00^a^	2 (1, 2)	0.51^a^
Rural	0 (0, 1)		1 (1, 1)		1 (0, 1)		1 (0, 1)		0 (0, 1)		0 (0, 0)		2 (1, 2)	
**Marital status**														
Married	1 (0, 1)	**<0.01** ^b^	1 (1, 1)	**<0.01** ^b^	1 (0, 2)	0.06^b^	1 (0, 1)	**<0.01** ^b^	0 (0, 1)	0.68^b^	0 (0, 0)	0.16^b^	2 (1, 2)	**0.04** ^b^
Single	0 (0, 1)		1 (1, 1)		1 (0, 2)		1 (0, 1)		0 (0, 1)		0 (0, 0)		2 (1, 2)	
Divorced/Widowed	1 (0, 2)		1 (1, 2)		1 (0, 2)		1 (0, 1)		0 (0, 1)		0 (0, 0)		2 (1, 2)	
**Educational status**														
Primary	0 (0, 1)	0.32^b^	1 (1, 2)	**0.01** ^b^	1 (0, 2)	**0.03** ^b^	0 (0, 1)	**<0.01** ^b^	0 (0, 1)	0.35^b^	0 (0, 0)	0.10^b^	2 (1, 2)	0.52^b^
Secondary	0 (0, 1)		1 (1, 1)		1 (0, 2)		1 (0, 1)		0 (0, 1)		0 (0, 0)		2 (1, 2)	
Higher	0 (0, 1)		1 (1, 1)		1 (0, 2)		1 (0, 1)		0 (0, 1)		0 (0, 0)		2 (1, 2)	
**Salary group**														
Low	0 (0, 1)	**<0.01** ^b^	1 (1, 1)	0.63^b^	1 (0, 2)	**0.03** ^b^	1 (0, 1)	**0.02** ^b^	0 (0, 1)	0.79^b^	0 (0, 0)	0.16^b^	2 (1, 2)	0.16^b^
Middle	0 (0, 1)		1 (1, 1)		1 (0, 2)		1 (0, 1)		0 (0, 1)		0 (0, 0)		2 (1, 2)	
High	1 (0, 2)		1 (1, 1)		1 (0, 1)		0 (0, 1)		0 (0, 1)		0 (0, 0)		2 (1, 2)	
**Physically active**														
Yes	0 (0, 1)	0.86^a^	1 (1, 1)	**0.02** ^a^	1 (0, 2)	0.07^a^	1 (0, 1)	0.67^a^	0 (0, 1)	0.51^a^	0 (0, 0)	1.00^a^	2 (1, 2)	0.95^a^
No	0 (0, 1)		1 (1, 1)		1 (0, 2)		1 (0, 1)		0 (0, 1)		0 (0, 0)		2 (1, 2)	
**Current smoker**														
Yes	0 (0, 1)	0.18^a^	1 (1, 1)	0.10^a^	1 (0, 2)	0.67^a^	1 (0, 1)	0.51^a^	0 (0, 1)	1.00^a^	0 (0, 0)	1.00^a^	2 (1, 2)	0.68^a^
No	0 (0, 1)		1 (1, 1)		1 (0, 2)		1 (0, 1)		0 (0, 1)		0 (0, 0)		2 (1, 2)	
**BMI group**														
Underweight	1 (0, 2)	**<0.01** ^b^	1 (1, 1)	**<0.01** ^b^	1 (0, 2)	0.31^b^	1 (0, 1)	**<0.01** ^b^	0 (0, 1)	0.62^b^	0 (0, 0)	0.49^b^	2 (1, 3)	0.20^b^
Normal	0 (0, 1)		1 (1, 1)		1 (0, 2)		1 (0, 1)		0 (0, 1)		0 (0, 0)		2 (1, 2)	
Overweight	1 (0, 2)		1 (1, 1)		1 (0, 2)		1 (0, 1)		0 (0, 1)		0 (0, 0)		2 (1, 2)	
Obese	1 (0, 2)		1 (1, 2)		1 (0, 2)		1 (0, 1)		0 (0, 1)		0 (0, 0)		2 (1, 2)	

*Bold values represent statistically significant associations p < 0.05; BMI (Body Mass Index)*.

a *Kolmogorov-Smirnov equality test*.

b *Kruskal-Wallis equality-of-populations rank test*.

### Associations of Quality of Sleep and Multimorbidity

Respondents who were in the poor quality of sleep group had a higher risk of multimorbidity compared with respondents in the good quality of sleep group (Table 3, Model 1, unadjusted OR = 2.17, 95% CI: 1.60, 2.96). The association of sleep quality and multimorbidity remained statistically significant after adjusting for demographics and socioeconomic factors (e.g., age, gender, educational, marital and salary status, geographical area, and residency) (Table 3). Specifically, participants who were in the poor quality of sleep tertile had 2.24 times higher odds of multimorbidity compared with participants in the good quality of sleep tertile, after adjusting for the other variables (Table 3, Model 2, adjusted OR = 2.24, 95% CI: 1.57, 3.20). Furthermore, individuals with moderate quality of sleep had a higher risk of having multimorbidity compared with those who were categorized in the good quality of sleep tertile (Table 3, Model 2, adjusted OR = 1.56, 95% CI: 1.05, 2.31). After the addition of lifestyle habits into the model, such as smoking and physical activity, the results indicated that the adjusted odds of multimorbidity were consistently higher and statistically significant for respondents in the moderate quality of sleep tertile (Table 3, Model 3, adjusted OR = 1.55, 95% CI: 1.04, 2.30) and the poor quality of sleep tertile compared with the good quality of sleep group (Table 3, Model 3, adjusted OR = 2.21, 95% CI: 1.55, 3.16). We also found statistically significant associations for age, gender, and middle-income group in Models 2 and 3 (Table 3). We tested the interactions terms between the quality of sleep tertiles and age, and also gender, and no statistically significant interactions (*p* > 0.05) were reported.

**Table 3:** Results of logistic regression on multimorbidity.

**Models**	**Model 1: Crude model^a^**	**Model 2: Crude model adjusted for demographic and socioeconomic characteristics^a^**	**Model 3: Crude model adjusted for demographic, socioeconomic and lifestyle characteristics^a^**
**Quality of sleep tertiles**			
Good	Ref	Ref	Ref
Moderate	1.26 (0.89, 1.78)	**1.56 (1.05, 2.31)**	**1.55 (1.04, 2.30)**
Poor	**2.17 (1.60, 2.96)**	**2.24 (1.57, 3.20)**	**2.21 (1.55, 3.16)**
**Age, per 1 year**	–	**1.06 (1.05, 1.08)**	**1.06 (1.05, 1.08)**
**Male gender**	–	**0.57 (0.41, 0.79)**	**0.54 (0.38, 0.77)**
**Educational status**			
Primary education	–	Ref	Ref
Secondary education	–	**0.47 (0.23, 0.94)**	0.51 (0.25, 1.03)
Higher education	–	0.53 (0.25, 1.13)	0.57 (0.27, 1.23)
**Marital status**			
Married	–	Ref	Ref
Single	–	1.14 (0.73, 1.79)	1.16 (0.73, 1.83)
Divorced/Widowed	–	1.19 (0.72, 1.98)	1.16 (0.70, 1.94)
**Salary group**			
Low	–	Ref	Ref
Middle	–	**1.65 (1.04, 2.62)**	**1.61 (1.01, 2.56)**
High	–	1.51 (0.88, 2.59)	1.53 (0.90, 2.63)
**Geographical area**			
Nicosia	–	Ref	Ref
Limassol	–	1.03 (0.71, 1.48)	1.04 (0.72, 1.50)
Larnaka	–	0.99 (0.61, 1.61)	1.03 (0.63, 1.69)
Paphos	–	0.78 (0.44, 1.39)	0.76 (0.42, 1.36)
Ammochostos	–	1.48 (0.67, 3.27)	1.48 (0.67, 3.27)
**Residency, rural/urban**	–	0.81 (0.54, 1.21)	0.81 (0.54, 1.22)
**Current smoking, yes/no**	–	–	1.29 (0.54, 1.22)
**Physical activity, yes/no**	–	–	0.83 (0.60, 1.14)

*Bold values represent statistically significant associations p < 0.05*.

a *Odds Ratio (OR), 95% Confidence Interval (C.I)*.

We also examined the association of poor vs. good sleepers with the probability of having multimorbidity after adjusting for possible confounders, such as age, gender, smoking, and physical activity status (Supplementary Table 3). We found that respondents who were in the poor quality of sleep group had a higher risk of multimorbidity compared with respondents in the good quality of sleep group in all models.

**Figure 1:**
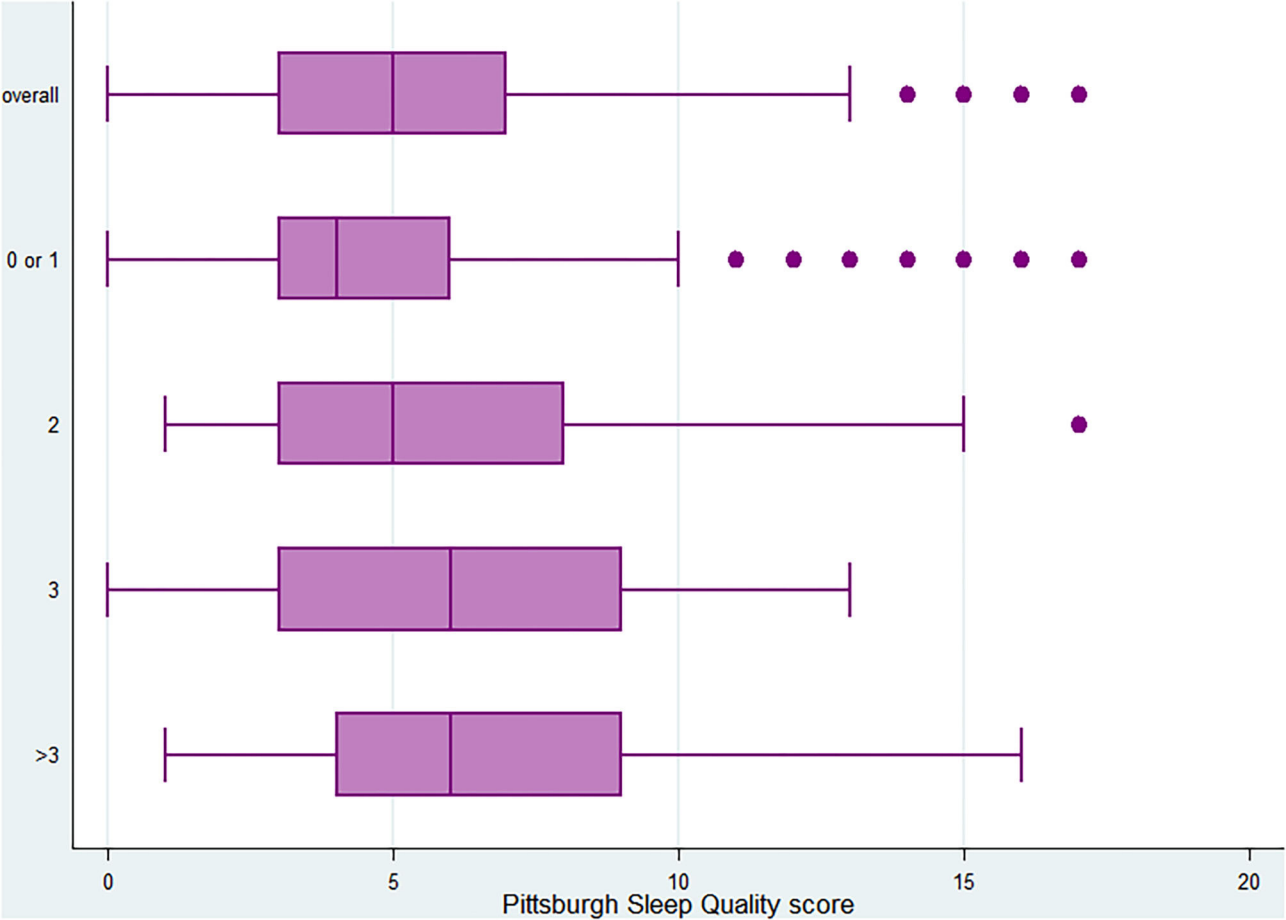
Distribution of pittsburgh sleep quality index (PSQI) score overall and by level of multimorbidity (0 or 1, 2, 3, and >3).

The median number of morbidities among the participants who were classified in the poor quality of sleep tertile was one (first quartile = 1 and third quartile = 2) whereas the median number of morbidities among the participants who were in the good and moderate quality of sleep tertiles was 0 (first quartile = 0 and third quartile = 1) and 1 (first quartile = 0 and third quartile = 1), respectively. Poorer quality of sleep, as indicated by the PSQI score, was observed among people with more than three morbidities, whereas better sleep was reported among people with 0 or one morbidity (Figure 1). Furthermore, people who were in the poor quality of sleep group had a higher risk of having two, three, or more than three morbidities, and the risk increases as the level of multimorbidity increases (Supplementary Table 4).

## Discussion

This population-based study of the general adult Cypriot population provided, to the best of our knowledge, the first evidence of the relationship between quality of sleep and multimorbidity. The results suggest that good quality of sleep is associated with lower odds of multimorbidity. Adopting a good quality pattern of sleep is beneficial and potentially helps reduce the risk of multimorbidity. This is an important public health result and supports the evidence available for the positive impact of a good quality of sleep on individual conditions.

The study found that the Cypriot population has a relatively good quality of sleep in general, although, almost one-third of the study population had a poor quality of sleep. We observed differences in the quality of sleep between men and women, between the residents of the five geographical areas of Cyprus, and among marital status categories. Specifically, women reported a poorer sleep quality compared to men, and this is consistent with other epidemiological studies (39, 40). This finding is possibly related to the fact that women have more sleep interruptions during several stages of their lives as well as the role of sex steroids (40). On the other hand, we did not identify any significant differences among educational and salary levels, between the residents of urban and rural regions, and based on smoking, or BMI. Furthermore, even though various epidemiological studies identified a high prevalence of sleep disturbances (7) and a low sleep quality (34) among older adults, in our study, we did not report poorer quality of sleep in people aged 65+ compared with people aged <65. Specifically, it was previously observed that there is a positive correlation between age and sleep quality scores of the elderly people and that as age increases the score of the quality of sleep decreases (34). In our study, 43 and 31% of the individuals aged 65 years old and older were in the good and poor quality of sleep tertile, respectively. The elderly people of our study have a high mean score on the quality of life assessment (EQ-5D) and almost 30% of them exercise regularly; thus these factors may play a role and explain why the poor quality of sleep in participants was not as prevalent in participants aged 65+. Other studies had previously suggested that quality of life (34) and exercise (35) may help improve the quality of sleep in elderly people.

Moreover, we reported that people who had a high annual income had a shorter sleep duration compared to those who had a low or a middle annual income. Even though this was an unexpected finding, a study that examined the sleep duration of 1,608 adolescents also found that individuals in the highest income group had shorter sleep duration than the lowest income group (41). Another study in the USA found that lower-income had an increased odd of both short and long sleep. In our study smaller duration of sleep was reported in individuals aged 45+ and given the fact that the participants with a higher salary were primarily those greater than 45 years old may also explain the observed association (42). We found that respondents who were classified in the poor quality of sleep group had a higher risk of multimorbidity compared with people who were in the good quality sleep group. Our finding supports previous research (12, 36, 43–45) that investigated the relationship between multimorbidity and specific sleep disorders, such as insomnia, or variations in sleep-related behavior, or sleep disturbances. We also observed that individuals who were in the poor quality sleep group had a higher risk of having two, three, or more than three morbidities, and the risk increases as the level of multimorbidity increases, which is in line with the study that investigated sleep duration and multimorbidity in Luxembourg (36). Moreover, our result agrees with the finding of a longitudinal study in Canada which reported that chronic diseases may be influenced by disrupted sleep (46). Finally, a recent study in China (47) reported that multimorbidity and poor sleep are associated overall in both genders and also among women separately (47).

However, it is not yet clear if the poor quality of sleep is a consequence of the presence of several chronic diseases in an individual or whether it leads to an increase in the number of chronic diseases in an individual. It has been reported that lack of sleep affects the endocrine pathways (48) and is associated with several chronic diseases, including cardio-metabolic and neurodegenerative diseases (23, 24). In fact, in our study, the most common combination of diseases among people with multimorbidity were diseases of the circulatory and endocrine systems (29). In addition, most of the people with multimorbidity who have an endocrine or circulatory disorder reported poor quality of sleep. This finding is in agreement with the evidence of the effects of bad sleep behaviors on several chronic diseases (34–36).

Our study has some limitations. First, the study is cross-sectional, which means that only associations between the groups of interest could be studied and not causal relationships. Moreover, all chronic diseases included in the questionnaire had equal weight in the calculation of multimorbidity since the severity of the disease is not measured. At the same time, the study has several strengths. It is a large population-based study of the Republic of Cyprus using a representative sample of both men and women from all ages (18+) and geographical areas. Furthermore, we collected detailed data on the participants including information on a large number of diseases.

In summary, the study provides evidence of an association between quality of sleep and multimorbidity. Specifically, we found that poor quality of sleep was associated with higher odds of multimorbidity. Other studies, such as intervention studies, should replicate these results and try to show causality, as poor quality of sleep may be a risk factor for the presence of two or more chronic conditions in an individual. Even though the association between quality of sleep and multimorbidity which was reported is not causal, it would be important to consider the effect of sleep on multimorbidity in any future prevention programs and practice guidelines.

## Conclusion

The findings suggest that the Cypriot population has a relatively good quality of sleep, in general, even though almost one-third of the population has a poor quality of sleep. A closer look at gender and marital status of Cypriots showed that women and married people had a poorer quality of sleep compared with men and individuals who are single, respectively. The study provides evidence of an association between quality of sleep and multimorbidity. The result suggests that poor quality of sleep is associated with higher odds of multimorbidity. Health education programs should be introduced to promote the positive effects of good sleep. Such programs could, for example, inform people, and especially young people, of the recommended number of hours of sleep, present the available evidence on sleep disorders and explain the symptoms of these disorders. In this way, people may recognize such symptoms and seek help. By tackling these disturbances which affect the quality of sleep, it can be improved, and the risk of multimorbidity can be reduced.

## Data Availability Statement

The raw data supporting the conclusions of this article will be made available by the authors, without undue reservation.

## Ethics Statement

The studies involving human participants were reviewed and approved by Cyprus National Bioethics Committee (CNBC) (EEBK EΠ 2018.01.123). Written informed consent for participation was not required for this study in accordance with the national legislation and the institutional requirements.

## Author Contributions

MK contributed to the conception and design of the study, the acquisition and analysis of data, and the writing of the first draft. CC and DP contributed to the formulation of the research hypothesis, design of the study, supervision, interpretation of the results, and critically editing the original draft. KM and MK contributed to the design of the study and in reviewing and editing the manuscript. All authors critically reviewed earlier drafts of the manuscript and approved the final version.

## Conflict of Interest

The authors declare that the research was conducted in the absence of any commercial or financial relationships that could be construed as a potential conflict of interest.

## Publisher's Note

All claims expressed in this article are solely those of the authors and do not necessarily represent those of their affiliated organizations, or those of the publisher, the editors and the reviewers. Any product that may be evaluated in this article, or claim that may be made by its manufacturer, is not guaranteed or endorsed by the publisher.

## Supplementary Material

Supplementary Table 1 Characteristics of participants by age group (18–24, 25–44, 45–64 and 65+ years old).

Supplementary Table 2 Demographics, socio-economic and lifestyle characteristics overall and by quality of sleep tertiles.

Supplementary Table 3 Results of Logistic Regression on multimorbidity.

Supplementary Table 4 Multinomial logistic regression of quality of sleep on multimorbidity level after accounting for age and sex (*N* = 1,140).
