# Supplementary material for: Quality of Sleep in the Cypriot Population and Its Association With Multimorbidity: A Cross-Sectional Study
Source: Front Public Health. 2021 Oct 29;9:693332. doi: 10.3389/fpubh.2021.693332 (PMC8585989; doi:10.3389/fpubh.2021.693332)
Supplement: Supplementary Table 1 — Characteristics of participants by age group (18–24, 25–44, 45–64 and 65+ years old). [file Table_1.docx]

| **Table S1.** Characteristics of participants by age group (18-24, 25-44, 45-64 and 65+ years old). | | | | | |
| --- | --- | --- | --- | --- | --- |
| **Characteristics** | **Age group** | | | | **p-value** |
|  | 18-24 | 25-44 | 45-64 | 65+ |  |
| **Physical activity** | | | | |  |
| No | 64 (39.0) | 247 (47.4) | 184 (59.0) | 96 (71.1) | **<0.01^a^** |
| Yes | 100 (61.0) | 274 (52.6) | 128 (41.0) | 39 (28.9) |  |
| **EQ-5D score** (mean ± SD) | 0.8 ± 0.2 | 0.8 ± 0.2 | 0.8 ± 0.2 | 0.7 ± 0.2 | **<.01**^b^ |
| Bold values represent p < 0.05; Abbreviations: SD, standard deviation; ^a^ Differences were evaluated by the chi-square test; ^b^Differences were evaluated by the t- test. | | | | | |
