# Supplementary material for: Quality of Sleep in the Cypriot Population and Its Association With Multimorbidity: A Cross-Sectional Study
Source: Front Public Health. 2021 Oct 29;9:693332. doi: 10.3389/fpubh.2021.693332 (PMC8585989; doi:10.3389/fpubh.2021.693332)
Supplement: Supplementary Table 2 — Demographics, socio-economic and lifestyle characteristics overall and by quality of sleep tertiles. [file Table_2.DOCX]

| **Table S2.** Baseline and medical characteristics by quality of sleep tertiles. | | | | |
| --- | --- | --- | --- | --- |
| **Characteristics** | **Overall^a^**  **(N=1,140)** | **Good^d^**  **(N=695)** | **Poor^d^**  **(N=445)** | **p-value** |
| **Quality of sleep score^a^** | 5 (3, 7) | 3 (2, 4) | 8 (6, 10) | **<.01**^b^ |
| **Number of morbidities** | 1 (0, 2) | 0 (0, 1) | 1 (0, 2) | **<.01**^b^ |
| **Age group** |  |  |  |  |
| *18-24* | 5 (3, 7) | 103 (61.7) | 64 (38.3) | 0.57^c^ |
| *25-44* | 5 (3, 7) | 328 (62.6) | 196 (37.4) |  |
| *45-64* | 5 (3, 7) | 188 (59.9) | 126 (40.1) |  |
| *65+* | 5 (3, 8) | 76 (56.3) | 59 (43.7) |  |
| **Sex** |  |  |  |  |
| *Male* | 4 (3, 6) | 375 (58.4) | 267 (41.6) | **0.04**^c^ |
| *Female* | 5 (3, 7) | 319 (64.2) | 178 (35.8) |  |
| **Geographical area** |  |  |  |  |
| *Nicosia* | 5 (3, 7) | 307 (62.3) | 186 (37.7) | **0.01**^c^ |
| *Limassol* | 5 (3, 7) | 191 (61.4) | 120 (38.6) |  |
| *Larnaka* | 5 (3, 7) | 108 (63.2) | 63 (36.8) |  |
| *Paphos* | 6 (4, 8) | 52 (46.0) | 61 (54.0) |  |
| *Ammochostos* | 5 (2, 6) | 35 (70.0) | 15 (30.0) |  |
| **Residency** |  |  |  |  |
| *Urban* | 5 (3, 7) | 515 (59.6) | 349 (40.4) | 0.14^c^ |
| *Rural* | 5 (3, 7) | 174 (64.7) | 95 (35.3) |  |
| **Marital status** |  |  |  |  |
| *Married* | 5 (3, 7) | 381 (61.8) | 235 (38.2) | 0.79^c^ |
| *Unmarried* | 5 (3, 7) | 256 (60.8) | 165 (39.2) |  |
| *Divorced/Widowed* | 5 (3, 8) | 56 (58.3) | 40 (41.7) |  |
| **Educational status** |  |  |  |  |
| *Primary* | 5 (3, 8) | 37 (56.1) | 29 (43.9) | 0.70^c^ |
| *Secondary* | 5 (3, 7) | 208 (61.5) | 130 (38.5) |  |
| *Higher* | 4 (1, 6) | 445 (61.0) | 284 (39.0) |  |
| **Salary group** |  |  |  |  |
| *Low* | 5 (3, 7) | 142 (58.9) | 99 (41.1) | 0.57^c^ |
| *Middle* | 5 (3, 7) | 340 (60.5) | 222 (39.5) |  |
| *High* | 4 (3, 7) | 207 (63.1) | 121 (36.9) |  |
| **Physically active** |  |  |  |  |
| *Yes* | 4 (3, 7) | 338 (57.2) | 253 (42.8) | <.01^c^ |
| *No* | 5 (3, 7) | 352 (65.1) | 189 (34.9) |  |
| **Current smoker** |  |  |  |  |
| *Yes* | 5 (3, 7) | 458 (62.6) | 273 (37.4) | 0.12^c^ |
| *No* | 5 (3, 7) | 233 (58.0) | 169 (42.0) |  |
| **BMI group** |  |  |  |  |
| *Underweight* | 5 (3, 7) | 26 (61.9) | 16 (38.1) | 0.18^c^ |
| *Normal* | 5 (3, 7) | 361 (63.9) | 204 (36.1) |  |
| *Overweight* | 5 (3, 8) | 205 (56.6) | 157 (43.4) |  |
| *Obese* | 5 (3, 7) | 93 (61.2) | 59 (38.8) |  |
| Bold values represent statistically significant associations p < 0.05; BMI (Body Mass Index);  ^a^ Median PSQI score (1^st^ quartile, 3^rd^ quartile)  ^b^ Kruskal-Wallis equality-of-populations rank test  ^c^ Pearson's chi-squared test  ^d^ N(%) | | | | |
