# Supplementary material for: Quality of Sleep in the Cypriot Population and Its Association With Multimorbidity: A Cross-Sectional Study
Source: Front Public Health. 2021 Oct 29;9:693332. doi: 10.3389/fpubh.2021.693332 (PMC8585989; doi:10.3389/fpubh.2021.693332)
Supplement: Supplementary Table 3 — Results of Logistic Regression on multimorbidity. [file Table_3.DOCX]

| **Table S3.** Crude and adjusted odds ratios of multimorbidity (n=1,140). | | | |
| --- | --- | --- | --- |
| **Models** | **Model 1:** Crude model^a^ | **Model 2:** Crude model adjusted for demographic and socioeconomic characteristics^a^ | **Model 3:** Crude model adjusted for demographic, socioeconomic and lifestyle characteristics^a^ |
| **Quality of sleep tertiles** |  |  |  |
| Good | *Ref* | *Ref* | *Ref* |
| Poor | **1.96 (1.50, 2.57)** | **1.99 (1.46, 2.71)** | **1.94 (1.42, 2.65)** |
| **Age, per 1 year** | - | **1.06 (1.05, 1.08)** | **1.06 (1.05, 1.08)** |
| **Male gender** | - | **0.56 (0.41, 0.78)** | **0.53 (0.37, 0.75)** |
| **Educational status** |  |  |  |
| Primary education | - | *Ref* | *Ref* |
| Secondary education | - | **0.47 (0.23, 0.94)** | 0.50 (0.25, 1.02) |
| Higher education | - | 0.52 (0.25, 1.12) | 0.56 (0.26, 1.21) |
| **Marital status** |  |  |  |
| Married | - | *Ref* | *Ref* |
| Unmarried | - | 1.12 (0.71, 1.76) | 1.16 (0.73, 1.83) |
| Divorced/Widowed | - | 1.19 (0.72, 1.97) | 1.16 (0.70, 1.93) |
| **Salary group** |  |  |  |
| Low | - | *Ref* | *Ref* |
| Middle | - | **1.62 (1.02, 2.57)** | **1.59 (1.01, 2.54)** |
| High | - | 1.47 (0.86, 2.51) | 1.50 (0.88, 2.57) |
| **Geographical area** |  |  |  |
| Nicosia | - | *Ref* | *Ref* |
| Limassol | - | 1.03 (0.71, 1.48) | 1.06 (0.74, 1.53) |
| Larnaka | - | 1.01 (0.62, 1.63) | 1.05 (0.64, 1.72) |
| Paphos | - | 0.80 (0.45, 1.42) | 0.77 (0.43, 1.38) |
| Ammochostos | - | 1.51 (0.68, 3.36) | 1.52 (0.68, 3.37) |
| **Residency, rural/urban** | - | 0.82 (0.55, 1.23) | 0.82 (0.54, 1.23) |
| **Current smoking, yes/no** | - | - | 1.31 (0.94, 1.82) |
| **Physical activity, yes/no** | - | - | 0.84 (0.61, 1.15) |
| Bold values represent statistically significant associations p < 0.05  ^a^ Odds Ratio (OR), 95% Confidence Interval (C.I) | | | |
