# Supplementary material for: Quality of Sleep in the Cypriot Population and Its Association With Multimorbidity: A Cross-Sectional Study
Source: Front Public Health. 2021 Oct 29;9:693332. doi: 10.3389/fpubh.2021.693332 (PMC8585989; doi:10.3389/fpubh.2021.693332)
Supplement: Supplementary Table 4 — Multinomial logistic regression of quality of sleep on multimorbidity level after accounting for age and sex (N = 1,140). [file Table_4.docx]

| **Table S4.** Multinomial logistic regression of quality of sleep on multimorbidity level after accounting for age and sex (N=1,140). | | | |
| --- | --- | --- | --- |
|  | Multimorbidity level (base outcome = 0 or 1 morbidities) | | |
|  | 2^a^ | 3^a^ | >3^a^ |
| Quality of sleep tertiles |  |  |  |
| Good | *Ref* | *Ref* | *Ref* |
| Moderate | 1.21 (0.72, 2.01) | 1.51 (0.76, 2.99) | **2.59 (1.36, 4.93)** |
| Poor | **1.91 (1.22, 2.99)** | **2.10 (1.15, 3.83)** | **3.00 (1.70, 5.27)** |
| Bold values represent statistically significant associations p < 0.05;  ^a^ Odds Ratio (OR), 95% Confidence Interval (C.I) | | | |
